# Application of Monoclonal Anti-Mycolate Antibodies in Serological Diagnosis of Tuberculosis

**DOI:** 10.3390/tropicalmed9110269

**Published:** 2024-11-06

**Authors:** Alma Truyts, Ilse Du Preez, Eldas M. Maesela, Manfred R. Scriba, Les Baillie, Arwyn T. Jones, Kevin J. Land, Jan A. Verschoor, Yolandy Lemmer

**Affiliations:** 1 Future Production: Chemicals, Council for Scientific and Industrial Research, Pretoria 0081, South Africa; almatruyts@gmail.com (A.T.); idpreez@csir.co.za (I.D.P.); mmaesela@csir.co.za (E.M.M.); mrscriba@csir.co.za (M.R.S.); 2 Department of Biochemistry, Genetics and Microbiology, University of Pretoria, Pretoria 0002, South Africa; jan.verschoor@up.ac.za; 3 School of Pharmacy and Pharmaceutical Sciences, Cardiff University, Cardiff CF10 3NB, UK; bailliel@cardiff.ac.uk (L.B.); jonesat@cardiff.ac.uk (A.T.J.); 4 Global Access Diagnostics, Thurleigh, Bedford MK44 2YA, UK; kevin.land@globalaccessdx.com; 5 Department of Electrical, Electronic and Computer Engineering, University of Pretoria, Pretoria 0002, South Africa

**Keywords:** tuberculosis, non-sputum-based diagnosis, point-of-care, lateral flow immunoassay, biomarker, mycolic acid

## Abstract

Patient loss to follow-up caused by centralised and expensive diagnostics that are reliant on sputum is a major obstacle in the fight to end tuberculosis. An affordable, non-sputum biomarker-based, point-of-care deployable test is needed to address this. Serum antibodies binding the mycobacterial cell wall lipids, mycolic acids, have shown promise as biomarkers for active tuberculosis. However, anti-lipid antibodies are of low affinity, making them difficult to detect in a lateral flow immunoassay—a technology widely deployed at the point-of-care. Previously, recombinant monoclonal anti-mycolate antibodies were developed and applied to characterise the antigenicity of mycolic acid. We now demonstrate that these anti-mycolate antibodies specifically detect hexane extracts of mycobacteria. Secondary antibody-mediated detection was applied to detect the displacement of the monoclonal mycolate antibodies by the anti-mycolic acid antibodies present in tuberculosis-positive guinea pig and human serum samples. These data establish proof-of-concept for a novel lateral flow immunoassay for tuberculosis provisionally named MALIA—mycolate antibody lateral flow immunoassay.

## 1. Introduction

Diagnosis of tuberculosis (TB) is still the biggest gap in the cascade to care for this condition [1,2,3]. To end TB, an affordable device is needed that uses non-invasive sampling and allows for decentralised diagnosis at the lowest level of the healthcare system [3,4]. The requirements for such tests were described in the 2014 target product profile (TPP) developed by the World Health Organization [5] that was recently updated [6]. The minimal requirements for a “non-sputum point-of-care (POC)” assay are 65% sensitivity, >98% specificity, less than 60 min for a test result and at a cost of less than United States dollar (USD) 4. For a “non-sputum near POC” assay, the minimal requirements are 75% sensitivity, >98% specificity, less than 60 min for a test result and at a cost of less than USD 6 [6]. 

While lateral flow immunoassay (LFIA) is an obvious technology to meet this TPP, several LFIAs for TB have been evaluated and found to be deficient [7,8,9]. Serological diagnosis is widely held to be unable to distinguish active disease from latent, asymptomatic infection [10]. TB diagnostic tests, in general, also face unreliability in immuno-compromised individuals [11], such as in human immunodeficiency virus (HIV) co-infected patients, who comprise 54% of people living with TB in South Africa and 18.5% in Africa [3]. 

LFIAs in use in the field do well when assessed against the ASSURED criteria [12]. The POC urine lipoarabinomannan (LAM) test has hinted at the impact that an LFIA for this TPP can have—reducing the relative risk of patient mortality in hospitalised HIV-positive patients by 17% [13]. However, the LAM test is limited to application in this group (HIV-positive patients with low cluster of differentiation (CD) 4 cell counts) only [14]—a category in which category it outperforms other tests [15]. Improvement in the sensitivity of this LFIA is the focus of many of the new products in the pipeline aimed at a non-sputum-based diagnostic [4]. 

Our approach is to obtain an effective LFIA using anti-lipid antigen antibodies as the biomarker analyte. Anti-lipid antibodies are produced early upon infection, decrease with successful treatment [16,17] and are likely not to be affected by previous vaccination against or exposure to TB [18,19]. Lipid antigens are presented via CD 1 restricted T-cells [20], and so anti-lipid antibodies are generated in a CD4 T-cell independent pathway [21] and are thus unaffected by HIV infection [22]. Mycolic acids (MA) are long-chain fatty acid components of mycobacterial cell walls that elicit an innate immune response [23]. Successful diagnosis of active TB by the detection of anti-MA antibodies has been shown using laboratory-bound techniques [18,24,25]. These techniques can compensate for the low affinity of anti-lipid antibodies but do not meet the REASSURED acronym of criteria [12] for deployment at the POC. 

The use of a purely lipoidal antigen in LFIA is unique, with the closest cases using glycolipids (such as LAM) in which the glycolic moiety can be used to attach the antigen to the membrane surface [26] and as the antigenic epitope [27]. The use of the MA glycolipid ‘cord factor’ to detect anti-MA antibodies is not specific enough to be of use [28]. 

Recombinant monoclonal antibodies were generated by selection of (variable) single chain fragments (scFvs) from a chicken-derived phage display immunoglobulin (Ig) gene library using a purified, natural mixture of MAs as antigen in enzyme linked immunosorbent assay (ELISA) [29]. Three clones (numbered 12, 16, and 18) were chosen for their stability during prolonged storage and for their varying binding specificities for MA subclasses. These antibody fragments were engineered into two types of bivalent IgY (chicken Ig) formats, one a theoretically flexible CH1-4 construct (CH denotes a constant heavy Ig domain) and the other a truncated and hypothetically more rigid CH2-4 type [29,30]. This resulted in six antibody constructs, called gallibodies. The gallibodies specifically detect MA in ELISAs and are transiently produced in Human Embryonic Kidney -293 cell cultures [29]. As ideal affinity purely mimics the anti-mycolate antibody biomarkers of TB in human patients, the way in which gallibodies recognise the mycolic acid antigen was investigated. We showed that they are unable to bind whole cell mycobacteria using an immunostaining technique, but that they are well able to detect the mycolic acid antigen in crude mycobacterial extracts.

**Figure 1:**
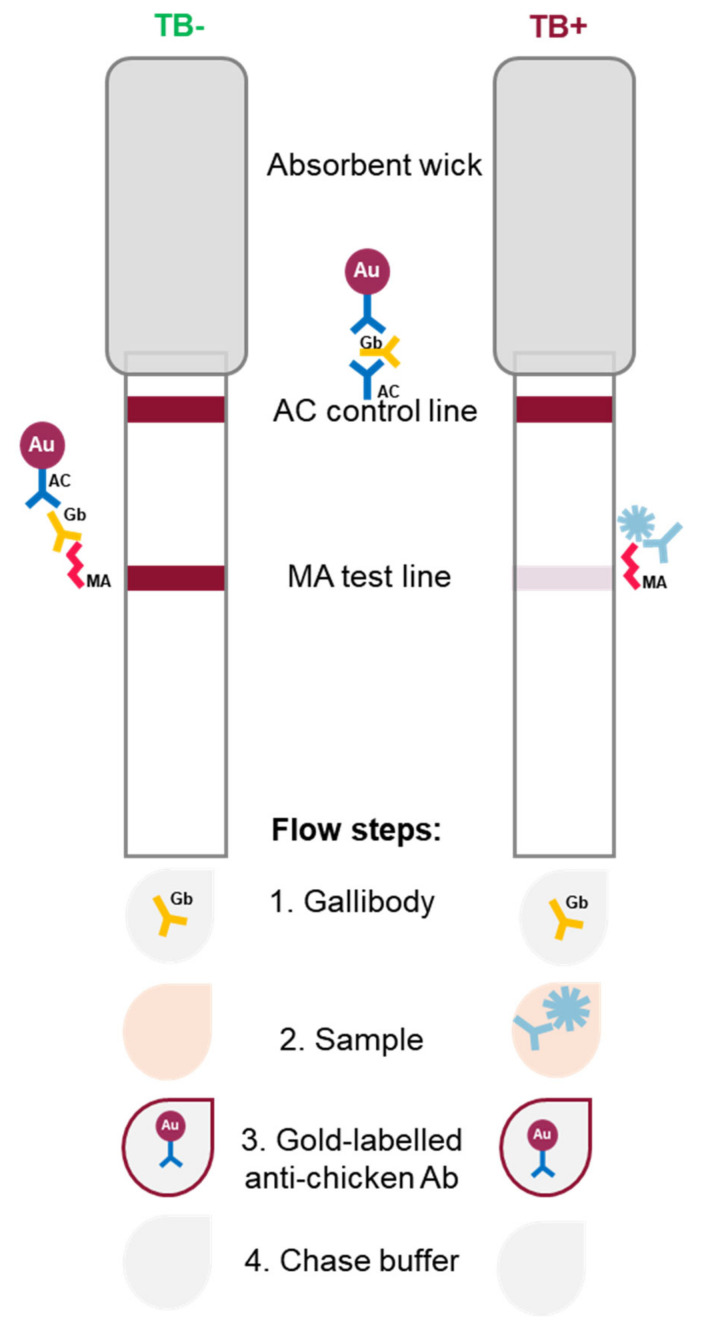
Mycolate Antibody Lateral Flow Immunoassay (MALIA) schematic. Monoclonal anti-mycolic acid (MA) antibody, ‘gallibody’ (Gb), binds to the MA antigen-coated test line and is bound by the anti-chicken (AC) antibody on the control line. In the case of TB-positive sera, biomarker anti-MA antibodies present in sera displace bound gallibody on the test line. Gold (Au)-labelled anti-chicken antibody binds gallibody on the test and control lines providing visible lines. TB—tuberculosis.

Here, we provide the first piece of evidence for the competitive detection of biomarker anti-MA antibodies in serum samples from laboratory-infected guinea pigs and human patients. This method of diagnosis of TB was envisioned by Verschoor and Beukes [31] upon the development of the first iteration of the gallibodies [32]. We present a competitive LFIA, which we tentatively labelled as MALIA (mycolate antibody lateral flow immunoassay). The principle of MALIA (Figure 1) is to detect the displacement of bound gallibodies by higher-affinity TB biomarker anti-MA antibodies in patient serum from the MA test line. This would produce a reduced/absent test line signal as an indication of active TB. Serum samples from patients without active TB (including latently infected or vaccinated patients) or uninfected guinea pigs should not displace any of the gallibodies bound to the test line, thereby leaving a dark red test line. A choice of six gallibodies is available to ensure optimal competitive displacement. 

The distinctive feature of MALIA is its wax-like antigen and the competitive (rather than direct) detection of the biomarker anti-MA antibodies in serum. These features differentiate this test from previously failed LFIAs for TB. The inherent low affinity of binding between antibody and MA is the main challenge to overcome in developing the proof-of-concept device. Continued efforts to realise MALIA as a POC test are justified by the current void of technology to provide a rapid, non-invasive screening test for continuous monitoring of people at risk of contracting TB.

## 2. Materials and Methods

### 2.1. Ethical Statement for All In Vitro and In Vivo Experiments

This study was conducted in accordance with the Declaration of Helsinki and approved by the Council for Scientific and Industrial Research (CSIR, South Africa) and University of Pretoria (UP) research ethics committees as follows CSIR 385 2021, 424 2023. UP 108/2014, 328/2021, 204/2022 and 352/2023. All animal experiments were conducted in accordance with the institutional and national guidelines and the relevant ethical approvals obtained prior to experimentation.

### 2.2. Reagents

Unless otherwise specified, salts and reagents were procured from Merck (Rahway, NJ, USA). The phosphate-buffered saline (PBS, composed of NaCl at 137 mM, KCl at 2.7 mM, Na_2_HPO_4_ at 10 mM and KH_2_PO_4_ at 1.8 mM) used was 0.1 M with a pH of 7.4. Commercial MA from *Mycobacterium tuberculosis* (Merck, USA, M4537) was used.

### 2.3. Mycobacterial ELISA

#### 2.3.1. Bacterial Culture

*Mycobacterium bovis* (BCG variant, NCTC 5692) was obtained from the UK Health Security Agency, and *M. tuberculosis* (H37Ra ATCC 25177) was grown in Middlebrook 7H9 medium (Difco, Becton Dickinson, Franklin Lakes, NJ, USA) supplemented with 0.5% *w*/*v* glycerol, 0.05% *w*/*v* tylaxopol and 10% *v*/*v* oleic-albumin-dextrose-catalase or albumin-dextrose-catalase (OADC/ADC) for 21–22 days. *Mycobacterium smegmatis* (NCTC 8159) and *Mycobacterium abscessus* (NCTC 13031) obtained from the UK Health Security Agency were grown in Luria Broth (Miller, Appleton, WI, USA) supplemented with 0.05% *w*/*v* tylaxopol for 4–6 days. Cultures were incubated without agitation at 37 °C and grown in Corning U-Shaped Cell Culture Flasks (15350591, Fischer, Wallingford, UK) containing at least 15 mL of media. All bacterial strains were cultured and handled under biosafety level 2 conditions.

*Escherichia coli* (25922) obtained from the UK Health Security Agency was grown in 25 mL Luria Broth (Miller) in a 50 mL falcon tube at 37 °C overnight. 

The cultures were harvested by centrifugation at 6000× *g* for 5 min, and the pellets were washed twice with sterile PBS and stored at −20 °C until use.

#### 2.3.2. Single-Cell Suspension

Single-cell suspensions of the mycobacteria were prepared from fresh or frozen bacterial pellets according to the protocol described by Leisching et al. [33] with minor modifications. Briefly, pellets were resuspended in 1 mL PBS and pipetted up and down through a 1 mL pipette tip 10 times (20 passes). This suspension was transferred into an insulin syringe (29G needle) with the plunger removed and carefully passed through the needle 10 times (19 passes). The syringed suspension was made up to a volume of 1.5 mL, and major clumps were allowed to settle for 10 min. The top 1.25 mL of the suspension was added to 1.75 mL of sterile PBS before filtering through a 5 µm pore size syringe filter (Sartorius Stedim, Göttingen, Germany). The optical density (OD) of the filtered suspension was measured using an Ultrospec 2100 pro spectrophotometer (Amersham Biosciences, Amersham, UK). This suspension was diluted if required to reflect an absorbance at 600 nm of less than 0.09 and taken to be approximately 10^8^ colony-forming units (CFU) per mL based on previous quantitation by dilution and plating. 

#### 2.3.3. Whole Bacteria ELISA

Single-cell suspensions of four species of mycobacteria and *E. coli* were coated in NUNC™ Maxisorp plate wells according to the protocol described by Bragg et al. [34]. Briefly, 100 µL of bacterial suspension at 10^7^ CFU/mL was air-dried in wells under a fan. Bacteria were fixed using 200 µL of 70% *v*/*v* methanol for 10 min. Methanol was flicked out and wells were air-dried before wrapping in parafilm and storing at 4 °C until use. As controls, 50 µL volume of purified MA in hexane or hexane only was also coated at 31.25 µg/mL. Hexane was allowed to evaporate at room temperature.

Plates were blocked for two hours, incubated with primary and then secondary antibodies for one hour each at room temperature with three washes between each step. For blocking, 300 µL of 1% (*w*/*v*) bioPLUS™ Hammarsten grade casein (bioWORLD, Visalia, CA, USA, 20960010 CAS: 9000-71-9) in PBS with a pH adjusted to 7 was used. To wash, the liquid was flicked out and the wells were washed three times after each step with 300 µL of PBS/0.1% (*v*/*v*) Sigma Tween^®^ 20 (wash buffer). For the primary antibody, 50 µL of gallibody 12-2 diluted to 0.04 mg/mL or a control rabbit polyclonal anti-*M. tuberculosis* antibody (Abcam Cat# ab905, London, UK, RRID: AB 306965) diluted 1:200 in the blocking buffer containing 0.1% *v*/*v* Tween 20 (dilution buffer) was used. For the secondary antibody, 50 µL goat anti-chicken Fc: HRP (Bio-Rad Cat# AAI29P, Hercules, CA, USA RRID: AB_323045) diluted 1:10 000 or goat anti-rabbit IgG (H+L): HRP (Thermo Fisher Scientific Cat# 32460, Waltham, MA, USA, RRID: AB_1185567) diluted 1:1000 was used. The reaction was developed by the addition of 50 µL of TMB (3, 3′, 5, 5′ tetramethylbenzidine, Invitrogen™, Carlsbad, CA, USA) substrate for 5 min before stopping the reaction with 50 µL of 2 M H_2_SO_4_. Absorbance was measured at 450 nm using a plate reader (Tecan Infinite M plex, Redwood City, CA, USA). 

### 2.4. Extract Preparation and ELISA

A 100 µL volume of the 10^8^ single-cell suspension was spread on agar plates. The mycobacterial strains were grown at 37 °C on Middlebrook 7H10 agar (Difco, Becton Dickinson, USA) supplemented with 0.5% *w*/*v* glycerol and 10% *v*/*v* OADC for 21–22 days for *M. bovis* and *M. tuberculosis* and 4–5 days for *M. smegmatis* and *M. abscessus.* The *E. coli* was grown on Luria agar (Miller) at 37 °C overnight. One *M. smegmatis* replicate (at 10^8^ concentration) was also grown on Luria agar for 4–5 days. 

To prepare crude hexane extracts, bacteria were scraped off the surface of agar plates using an inoculating loop until most of the bacteria had been removed. Bacteria on the inoculating loop were rinsed off into 500 µL of PBS in an Eppendorf tube, which was centrifuged at 6000× *g* for 5 min to collect the pellet of the bacteria. The pellets were resuspended in 1 mL hexane and transferred to glass vials with solvent-resistant caps. Vials were placed on a rocker at room temperature overnight. To harvest, the extracts were transferred to Eppendorf tubes and centrifuged at 6000× *g* for 5 min, and the hexane fraction was carefully pipetted into clean vials or tubes. Clean extracts were diluted 10-fold (100 µL extract added to 900 µL hexane). 

The ELISA was carried out in a similar fashion to that described by Ranchod, et al. [29]. NUNC™ Maxisorp plate wells were coated with 50 µL of the undiluted and diluted extracts. A serial dilution of purified MA in hexane was also coated at the following concentrations (µg/mL): 62.5, 3.125 (1 in 20, dilution of 62.5), 1.041 (1 in 3, dilution of 3.125) and 0.781 (1 in 4, dilution of 3.125). Hexane was allowed to evaporate at room temperature. 

The ELISA was carried out for the whole bacteria with the following changes: For blocking 300 µL 2% (*w*/*v*) bovine serum albumin (BSA) fraction V, heat shock, fatty acid-free (Merck, 03117057001) in PBS was used. For the primary antibody, 50 µL of gallibody diluted to 0.04 mg/mL in the BSA-blocking buffer containing 0.1% *v*/*v* Tween 20 (dilution buffer) was used. 

One confluent plate grown for the same amount of time from single-cell suspensions at similar concentrations was used for three replicate extracts. The figures were created using R [35] and R studio [36] software incorporating the ‘tidyverse’ [37] and ‘readxl’ [38] packages. The code is available upon request. 

### 2.5. LFIA

2-(N-morpholino) ethane sulfonic acid (MES) buffer was used at 0.01 M pH 7. Membrane-blocking solution used was the Invitrogen™ membrane-blocking solution (000105, ThermoFischer Scientific USA), which is a proprietary mixture, including BSA, goat Ig and Tween 20 in a Tris buffer.

#### 2.5.1. Colloidal Gold Conjugation

A 40 nm colloidal gold suspension (from DCNDX, Carlsbad, CA, USA; or Abcam, UK) supplied at an OD of 1 was pH-adjusted to 9 using dibasic sodium carbonate (Fluka, London, UK). Goat anti-chicken antibody (Bio-Rad Cat# AAI29, RRID:AB_323047) supplied (at 1 mg/mL) in PBS was diluted in 1/5th of the required gold volume in the MES buffer. The diluted antibody was added directly to the pH-adjusted colloidal gold to a concentration of 0.015 mg/mL in the OD 1 gold suspension and the tube inverted. After 30 min, a volume equivalent to the colloidal gold of BSA (Hyclone™, fraction V from GE Healthcare, Chicago, IL, USA) at 21 mg/mL in MES buffer was added. After 90 min, the gold conjugate was centrifuged at 13,000× *g* for 15 min and the supernatant discarded. The pellet was resuspended in 1/10th of the original gold volume of 15% (*w*/*v*) sucrose in the MES buffer. 

#### 2.5.2. LFIA Manufacture and Testing

Nitrocellulose membrane (CN95, Sartorius Stedim Biotech, Göttingen, Germany) 25 mm in width was affixed onto 30 cm long adhesive plastic backing cards (Kenosha C.V., Amstelveen, The Netherlands). The test and control lines were dispensed using a BioDot 360 printer. For the control line, goat anti-chicken antibody (Bio-Rad Cat# AAI29, RRID: AB_323047) was striped 5 mm below the top of the membrane at 0.25 mg/mL at 2 µL/cm in PBS. For the MA (dissolved in hexane) test lines, the normal tubing was replaced with solvent-compatible polytetrafluoroethylene (PTFE) tubing from the reservoir up to the micro tube. Three coats of MA were striped at 0.5 mg/mL at 2 µL/cm in hexane down the centre of the membrane. Coated membranes were dried for 5 min in an oven with a fan at 37 °C.

Membrane-blocking solution was pipetted onto the membrane until the entire membrane was wet and the excess liquid shaken off before blotting the membranes using a double layer of paper roller towel. The membranes were dried for 5 min in an oven with a fan at 37 °C. Cellulose fibre wicks were affixed with a 2 mm overlap at the top of the nitrocellulose membrane on the adhesive backing card. Tests were cut into strips of approximately 0.5 cm wide. 

The gallibodies were expressed in human embryonic kidney cells, purified from the culture media, as described in [29], and concentrated in the MES buffer. Gallibodies were stored at −20 °C until use.

Gallibody diluted in membrane blocker (50 µL) was flowed until completely absorbed; thereafter, 50 µL of the serum diluted 1 in 10 in membrane blocker containing a rheumatoid factor interference blocker (RFIB, 1 mg/mL, A20100009 Molecular Depot, San Diego, CA, USA) was flowed until absorbed. Control tests without serum were also performed. A 50 µL volume of membrane blocker and 3 µL of anti-chicken conjugate was added into the bottom of the tubes and flowed for a further 15–20 min. To reduce the background binding of the conjugate onto the nitrocellulose membrane, another 50 µL of the membrane blocker was flowed on the tests. 

Still-wet tests were laid face down to scan using an HP Scanjet G2410 Flatbed Scanner. Scans were edited using the accompanying HP Solution Centre software (version 1.1) as follows: cropped to size and “lighten/darken” parameters changed to highlights −100, shadows −100, midtones −100 and gamma 1.6. Files were saved as TIFF or JPEG file formats. Tests were dried in position overnight and the scan repeated. A single scan of all the tests in an experiment was performed and used for all further edits/quantitation. Cropped images of the test sections from these scans are presented in the figures.

### 2.6. Guinea Pig Serum 

Historical serum samples from a previous study involving TB-infected guinea pig animals were used. Briefly, female outbred Dunkin–Hartley guinea pigs (~300 g) were housed under barrier conditions in a biosafety level III animal facility. For infection, *M. tuberculosis* H37Rv (ATCC) was grown in Middlebrook 7H9 supplemented with 0.5% Tween 80, 0.2% glycerol and 10% OADC and stored in vials frozen at −70 °C until use. The animals were thereafter exposed to an aerosol of *M. tuberculosis* H37Rv by using a mesh nebuliser to deliver an inoculum of 20–30 bacteria into the guinea pig lungs. Blood was collected using the intra-cardiac method (~1 mL) from all the animals at Day 0 and at day of termination into clot activator tubes. TB-positive animals were confirmed by homogenising the lungs and spleen in PBS, plating serial dilutions of these on 7H11 agar plates and enumerating the viable bacteria by CFU counts. Colonies were confirmed as *M. tuberculosis* by (polymerase chain reaction) PCR. The sera from the uninfected (TB-negative) animals were pooled. Sera were used from three TB-positive animals (denoted x, y and z) that were euthanised 73 days post-infection, and CFU counts are given in Table S1. Sera were securely stored at −20 °C until use. 

### 2.7. Human Serum

To strengthen the proof-of-concept data, a pair of human serum samples (one negative and one positive for TB) were obtained as a kind gift from MARTI-TBD (South Africa). MARTI-TBD are developing a biosensor-based TB screening test using anti-MA antibodies biomarkers of active TB (personal communication). Whole blood samples were collected before treatment commencement from a culture-confirmed presumptive TB patient and a TB-negative sample from a healthy volunteer with no history or symptoms of TB. TB was diagnosed using the mycobacterial growth indicator tube system (the gold standard diagnostic) and ruled out in the TB-negative sample using the interferon-gamma release assay. The blood samples were centrifuged and the resultant serum aliquoted and stored at −80 °C until use. The sera were tested in MALIA in the same way as the guinea pig sera.

### 2.8. Data Analysis and LFIA Signal Quantitation

For the ELISA assays, wells were processed in triplicate, and *n* = 3, with the repeats presented separately. For the LFIA analysis, the scanned test signal intensity was analysed using the open-source image analysis package Fiji [39] (Fiji is based on ImageJ). This has been shown to be a comparable method to a commercial reader, with this method adapted from the principles described in Smith et al. 2019 [40]. The brightness and contrast of the image were adjusted to the ‘auto’ parameters (software optimised) using the software. Test signal lines were selected as the region of interest measured, taking special care to avoid the edge of the test and any pen marks, etc. This is shown in Supplementary Figure S1. In Fiji, the signal intensity of colour images is measured by averaging the red, green and blue pixels to brightness values for the selected region of interest, which is called the ‘average grey value’ [41]. Pure black would be the minimum value and white the maximum. The ‘average grey value’ of the selected area was measured and used for further comparison. 

The test line intensity of the TB-positive and -negative samples was normalised by dividing them by the average of the test line intensity obtained in the absence of serum. Duplicate tests were carried out for each condition. The available volume of guinea pig and human serum limited the number of replicate tests performed. This work was to determine whether displacement of the gallibody by serum antibodies to MA would occur and establish the basic protocol requirements to achieve this. Figures were generated using Tableau software (version 24.2), and duplicates are presented separately. 

## 3. Results

### 3.1. Whole Mycobacterial Detection via MA Antigen

**Figure 2:**
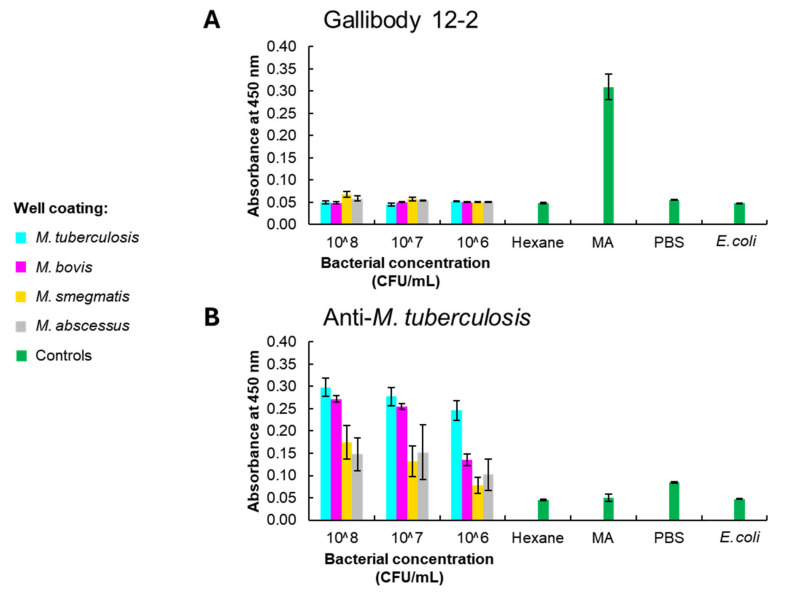
Whole-cell detection using gallibody. Single-cell suspensions of four mycobacterial species and *E. coli* were dried in triplicate wells, fixed in 70% methanol and blocked with 1% casein (in PBS at pH 7). Wells were probed with gallibody 12-2 at 0.04 mg/mL (**A**) or a polyclonal rabbit anti-*M. tuberculosis* (ab905) at 1:200 dilution (**B**). Binding was detected using relevant secondary antibodies conjugated to horseradish peroxidase. Average absorbance at 450 nm (bar heights) and standard deviation (error bars) with *n* = 3 shown. MA – mycolic acid; PBS – phosphate buffered saline CFU—colony-forming units.

The binding of the recombinant anti-MA ‘gallibodies’ to whole bacteria was investigated using a whole cell ELISA performed as described by Bragg et al. [34]. Fixed single-cell suspensions of four species of mycobacteria were probed with the most sensitive [29] gallibody of the six available, namely gallibody 12-2. This was compared to binding by a control polyclonal anti-*M. tuberculosis* (ab905) antibody procured from Abcam, UK. The results are shown in Figure 2. 

Gallibody 12-2 strongly gave high readings against the immobilised MA but not against the fixed, whole bacteria (Figure 2A). The anti-*M. tuberculosis* antiserum is recommended as “suitable for immunohistochemistry applications” by the manufacturer [42]. It specifically bound the coated mycobacteria (rather than the *E. coli*), with a marked preference for *M. tuberculosis* and *M. bovis* (Figure 2B). Cross-reactivity with other mycobacterial species was predicted by the manufacturer [42]. 

### 3.2. Characterisation of Gallibody Binding Specificities

MA subclass composition patterns are characteristic of mycobacterium species, for example, alpha-, keto- and methoxy MA in *M. tuberculosis* and *M. bovis* [43]. Previous characterisation of the gallibodies demonstrated their MA subclass specificity using the phage-displayed scFvs and chemically synthetic, stereochemical controlled preparations of alpha-, keto-, and methoxy MA [30]. The clone 12 scFvs bound all three subclasses, clone 16 scFvs bound trans-keto and methoxy MA clone, and clone 18 scFvs bound only the methoxy MA [29]. We investigated whether the subclass specificity of the gallibodies translated to mycobacterial species specificity. Crude hexane extracts of four mycobacteria species were probed with all six gallibodies alongside an *E. coli* extract in ELISA. 

Whole, live bacteria grown on agar were scraped off the plates and pelleted. Pellets were incubated in hexane overnight, and bacteria were removed by centrifugation. The hexane extracts (and one in ten dilutions of these) were used to coat NUNC™ Maxisorp plates for ELISA. Triplicate wells of each extract/control were coated. Qualitative thin layer chromatography (TLC) analysis of the extracts is provided in the Supplementary Information. The mycobacterial extracts showed spots with the same or similar retention factor to the control MA. Varying concentrations of MA in each replicate extract were observed.

**Figure 3:**
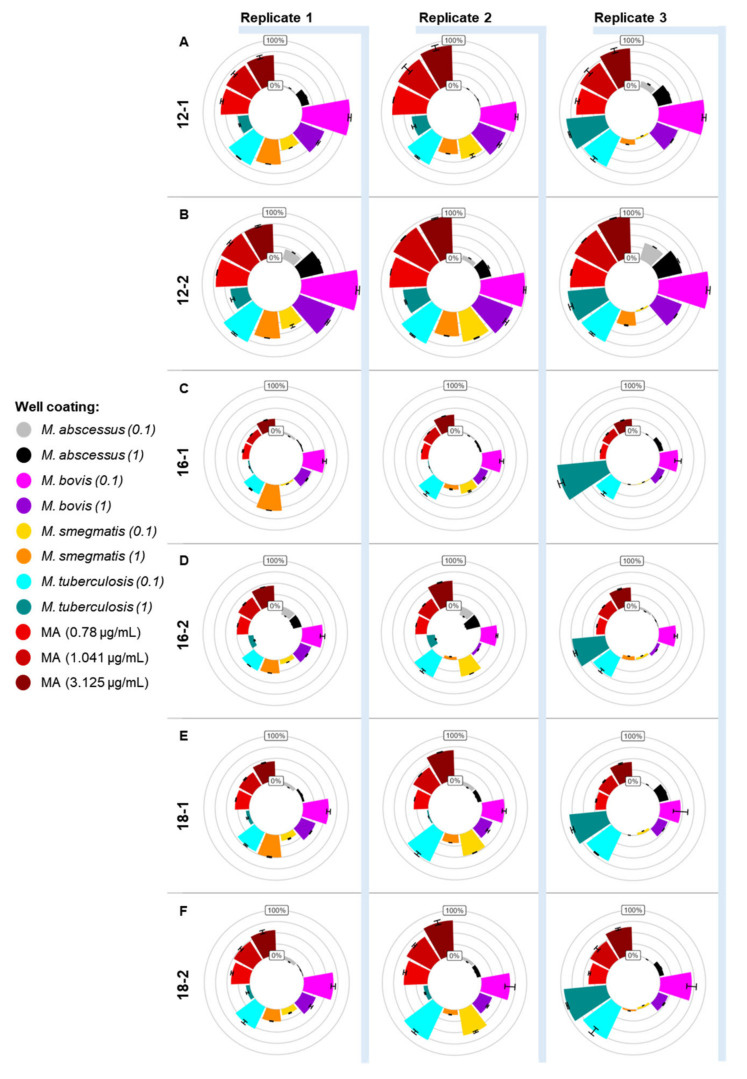
Mycobacterial species specificity of gallibodies. Crude hexane extracts (1× and 0.1×) of 4 species of mycobacteria as well as *E. coli* were probed with all 6 gallibodies (**A**–**F**) in ELISA. Three replicate extracts (shown separately) and controls were coated in triplicate wells (technical repeats). Technical replicates averaged for each coating; standard deviation presented as error bars. Data are represented as a percentage of the signal obtained for 62.5 µg/mL commercially purified MA (100%) with the average of the *E. coli* extract and the hexane-only signals as the inner 0% circle. Three further concentrations of purified MA (0.78, 1.041 and 3.125 µg/mL) were also probed for comparison. Key is listed clockwise from 12 o’clock. MA—mycolic acid.

The ELISA signal obtained for the *E. coli* extract and the hexane-only coated wells was averaged and subtracted from the other signals for each gallibody to demonstrate the mycobacterial specificity of the gallibodies. Signals are presented as a percentage of the signal obtained by each gallibody on the purified MA coated at 62.5 µg/mL. Results for the three replicates are shown separately in Figure 3. 

In Figure 3, all the gallibodies consistently bound the commercial MA (red) and mycobacterial extracts preferentially to the average of the *E. coli* extract and hexane-only signals (inner circle) demonstrating the specificity of the gallibodies. Preferred detection of the *M. tuberculosis* (cyan) and *M. bovis* (purple) extracts was shown (higher signal strength compared to other extracts) for all the gallibodies. 

All the gallibodies also bound at least one of the concentrations of the *M. smegmatis* extract (orange) for at least two of the replicates. The *M. smegmatis* extract used for replicate 3 (far right) was generated from bacteria grown on Luria agar rather than the 7H10 agar used for the other extracts. No visible MA concurrent spot was observed for the TLC analysis of replicate 3 of the *M. smegmatis* extract (S1). Luria agar does not provide the same raw materials (such as oleic acid) that allow for the rapid biosynthesis of MAs (within 4 days of incubation), providing a possible explanation for the lack of MA in this extract. 

The *M. abscessus* extracts (grey and black) were consistently weakly detected by 12-2, the most sensitive and least selective of the gallibodies. The 12 scFv cloned into the larger, theoretically more flexible scaffold, as 12-1 also bound the *M. abscessus* extract, but less well. Gallibodies 16-1, 18-1 and 18-2 weakly bound the undiluted *M. abscessus* extract for replicate 3. The 16-2 gallibody did not show any binding of the *M. abscessus* extract. As the gallibody that did not bind to any *M. abscessus* extract, 16-2 was the most specific, followed by 16-1, 18-2 and 18-1 (increasing degrees of binding to *M. abscessus* extract).

Interestingly, all the gallibodies showed increased binding to the diluted extracts of *M. bovis* (lighter purple), replicates 1 and 2 of *M. tuberculosis* (lighter cyan) and replicate 2 of *M. smegmatis* (yellow). This dilution-induced antigenicity is not observed upon dilution of the purified MA in the concentration range tested (red). This is likely dependent on the starting concentration of the extract (before dilution), which would vary between replicates. The gallibody least sensitive to reduction in the coated MA concentration (red) was 12-2, followed by 12-1, 18-2, 18-1, 16-2 and 16-1.

### 3.3. Displacement of Bound Gallibodies by Guinea Pig Antibodies to MA

Laboratory TB-infected guinea pig and healthy control sera were used for the initial proof of concept of the MALIA diagnostic device. Sera from terminally bled, pathogen-free, outbred Dunkin–Hartley guinea pigs were used to demonstrate the reduction in the MA test signal by displacement of the gallibodies in the samples. Sera from uninfected guinea pigs were pooled and used as the TB-negative sample. The TB-positive animals were infected via aerosol inhalation and euthanised 73 days post-infection. TB infection of the animals was confirmed by solid culture of homogenised lung and spleen tissue with colonies confirmed as *M. tuberculosis* by PCR. 

**Figure 4:**
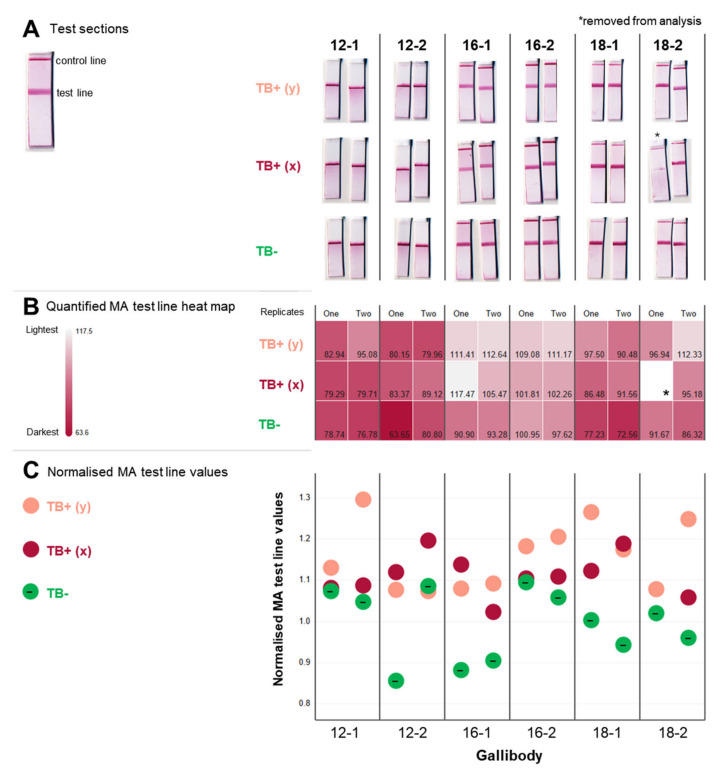
Reduction in MA signal by serum samples from TB-positive guinea pigs. (**A**) Scanned test sections. Gallibodies at 0.06 mg/mL diluted in membrane blocker was flowed on tests striped with anti-chicken IgG (Fc) antibody (top line) at 0.25 mg/mL and mycolic acid (bottom line) at 3 × 0.5 mg/mL for ~15 min. Subsequently, 50 µL of 10% guinea pig serum (pooled serum from TB-negative animals—‘TB−’, two TB-positive animals—‘TB+ (x)’ and ‘TB+ (y)’ diluted in membrane blocker containing a rheumatoid factor interference blocker (1 mg/mL) was flowed until absorbed, followed by 50 µL of membrane blocker containing 3 µL of anti-chicken-gold conjugate. Cropped scans of the test section of duplicate tests are shown; (**B**) heatmap of MA test line intensity from tests in (**A**). Fiji software analysis was performed on the scanned tests to quantify the intensity of the mycolic acid (MA) test line. The average of the red, green and blue pixels for the selected region of interest (in the test line) were used to generate the heatmap and are given in the bottom right of each block; (**C**) normalised MA test line intensity values. MA test line intensity (as in (**B**)) of individual replicate tests was normalised to the average of the MA test line intensity obtained using buffer only (no serum) for each gallibody. Buffer-only tests and test line intensity values are shown in Supplementary Figure S3 and Table S3. TB—tuberculosis.

An increased titre of IgM rheumatoid factor has been reported in active TB patients compared to healthy controls [44] with interference in commercial immunoassays reported [45], which we wished to preclude. In Figure 4, a 10% (*v*/*v*) serum solution containing a rheumatoid factor interference blocker (Molecular Depot, USA) was flowed on the test following the application of gallibody.

The test line signals (Figure 4A) were quantified using Fiji image analysis software [39] to allow for an objective comparison of test line signal intensity. The method used was adapted from that used by Smith et al. [40], which was shown to be comparable in quality to a commercial reader. The quantified test line signals of replicate tests are presented in a heatmap (Figure 4B) and normalised to the average of test line signals obtained in the absence of serum for each gallibody (Figure 4C). Control tests performed without serum and using BSA instead of gallibody are shown in Figure S3 with quantified test line signals in Table S3. 

Visible reduction in the MA test line signal by TB-positive samples compared to TB-negative samples especially when using gallibodies 16-1, 18-1 and 18-2 (Figure 4A) provides proof of the MALIA diagnostic concept. When the signal intensities were quantified (Figure 4B,C), the test signal intensities for the TB-negative samples (green/minus labelled) were shown to be consistently higher (darker) than the test line signals for the TB-positive samples (red and pink). 

The 16-1 gallibody gave the biggest difference between TB-positive and negative samples (Figure 4C) and clear control lines (top lines, Figure 4A). Gallibodies 12-2, 12-1 and 18-1 bound too strongly to the MA test line for good displacement (12-1 and 12-2), causing a sub-optimal anti-chicken control line signal (18-1, 12-1 and 12-2). 

**Figure 5:**
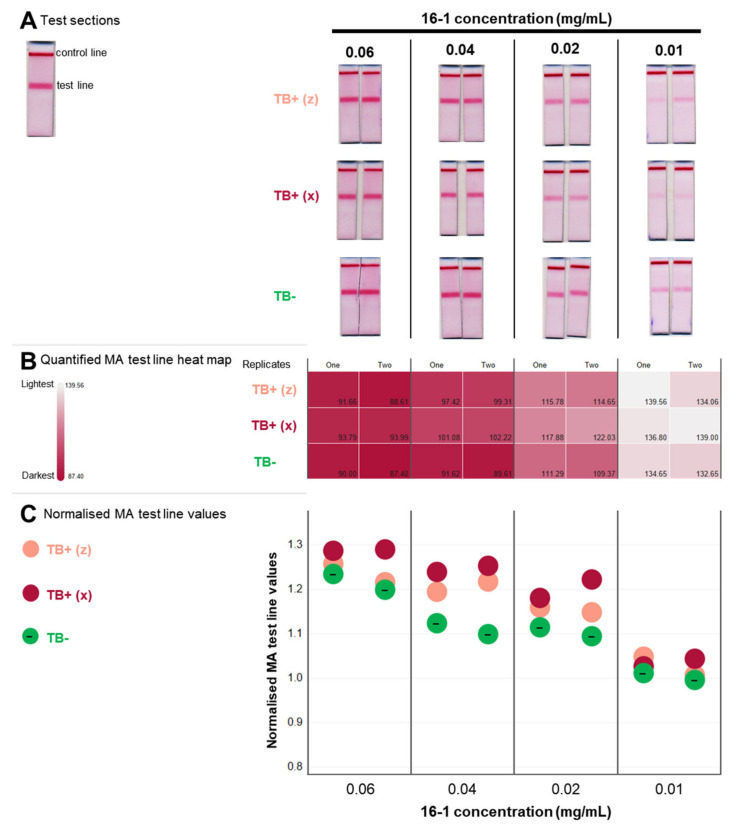
Titration of gallibody 16-1 concentration for optimal detection of anti-MA antibody in TB-positive guinea pig sera. (**A**) Gallibody 16-1 at 0.06, 0.04, 0.02 and 0.01 mg/mL diluted in membrane blocker was flowed on tests striped with anti-chicken IgG (Fc) antibody (top line) at 0.25 mg/mL and mycolic acid (bottom line) at 3 × 0.5 mg/mL for ~15 min. Subsequently, 50 µL of 10% guinea pig serum (pooled serum from TB-negative animals—‘TB−’, and two TB-positive animals denoted x and z—‘TB+ (x)’ and ‘TB+ (z)’ diluted in membrane blocker containing a rheumatoid factor inhibition blocker (1 mg/mL) was flowed until absorbed, followed by 50 µL of membrane blocker containing 3 µL of anti-chicken-gold conjugate. Cropped scans of the test section of duplicate tests are shown; (**B**) heatmap of MA test line intensity from tests in (**A**). FIJI software analysis was performed on the scanned tests to quantify the intensity of the mycolic acid (MA) test line. The average of the red, green and blue pixels for the selected region of interest (in the test line) were used to generate the heatmap and are given in the bottom right of each block; (**C**) normalised MA test line intensity values. MA test line intensity (as in (**B**)) of individual replicate tests was normalised to the average of the MA test line intensity obtained using buffer only (no serum) for each concentration. Buffer-only tests and test line intensity values are shown in Supplementary Figure S4 and Table S4. TB—tuberculosis.

Gallibody 16-1 was selected to investigate the effect of the concentration of the gallibody on the displacement (Figure 5).

The effect of titration of the concentration of gallibody to be displaced by serum anti-MA antibodies is shown in Figure 5 (quantified in Figure 5B,C). As expected, the overall MA signals reduce with reducing gallibody concentration. The best distinction between the TB-positive and TB-negative sera was observed at a gallibody concentration of 0.04 mg/mL, with the next highest at 0.02 mg/mL (Figure 5C). The poorest distinction between the TB-positive and TB-negative sera happened with the maximum and minimum gallibody concentrations tested. 

Due to the paucity of the samples, replicated experiments could not be performed to strengthen these data, especially the numerical conversion of the signal intensities. This limitation prevented a statistical interpretation of the data observed (Figure 5C). Nevertheless, the pattern observed strongly indicates 0.04 and 0.02 mg/mL as the most useful gallibody concentrations to distinguish between TB-positive and TB-negative serum samples in MALIA for further testing.

### 3.4. Reduction in MA Signal by a TB-Positive Patient’s Serum Sample

**Figure 6:**
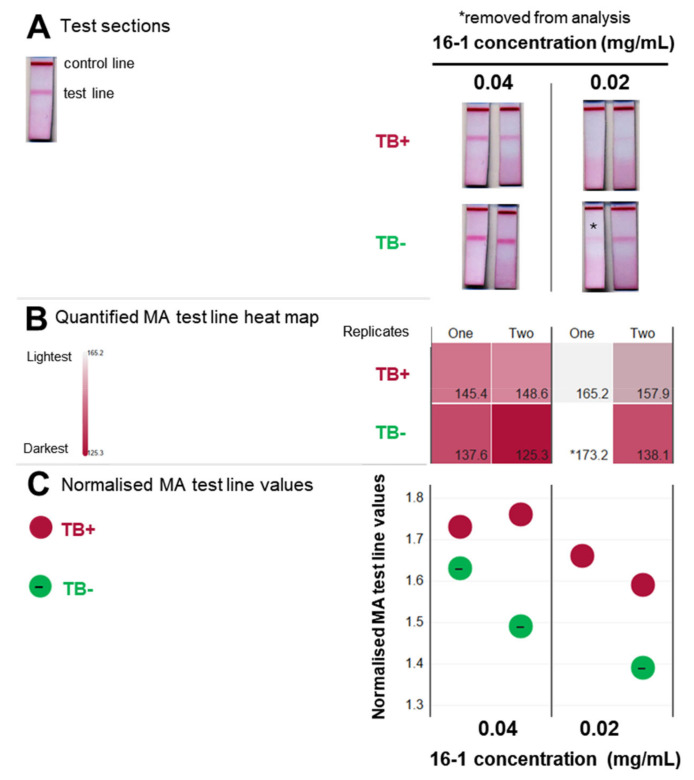
MALIA diagnostic differentiates TB-positive and -negative human serum samples. (**A**) Gallibody 16-1 at 0.04 and 0.02 mg/mL diluted in membrane blocker was flowed on tests striped with anti-chicken IgG (Fc) antibody (top line) at 0.25 mg/mL and mycolic acid (bottom line) at 3 × 0.5 mg/mL for ~15 min. Subsequently, 50 µL of 10% human serum (TB− or TB+) diluted in membrane blocker containing a rheumatoid factor inhibition blocker (1 mg/mL) was flowed until absorbed. Tests were then developed with 50 µL of membrane blocker containing 3 µL of anti-chicken-gold conjugate. Cropped scans of the test section of duplicate tests are shown. (**B**) Heatmap of MA test line intensity from tests in (**A**). FIJI software analysis was performed on the scanned tests to quantify the intensity of the mycolic acid (MA) test line. The average of the red, green and blue pixels for the selected region of interest (in the test line) were used to generate the heatmap and are given in the bottom right of each block; (**C**) normalised MA test line intensity values. MA test line intensity (as in (**B**)) of individual replicate tests was normalised to the average of the MA test line intensity obtained using buffer only (no serum) for each concentration. Buffer-only tests and test line intensity values are shown in Supplementary Figure S5 and Table S5. * Test removed from analysis due to scan artefact. TB—tuberculosis.

The use of the guinea pig model of experimental TB infection in a controlled environment is an idealised system for testing the proof of concept of MALIA. To confirm whether the MALIA test would also distinguish between TB-positive and TB-negative humans, we obtained two human serum samples (a confirmed TB-positive and TB-negative) as a kind gift from MARTI-TBD (South Africa) to test in MALIA. The human sera were applied and compared in the same way as the guinea pig sera (Figure 6).

Clear reduction in the test line signal by the human TB-positive sample was observed at both concentrations of gallibody (Figure 6A). This result provided a positive proof-of-concept for the diagnostic principle of MALIA to distinguish between serum samples from TB-positive and healthy humans. A scan artefact is evident for the TB-negative test at 0.02 mg/mL gallibody with a lighter test line value obtained (Figure 6B) for it than any other in the set, which is not the case when comparing by eye.

## 4. Discussion

An accurate, non-sputum-based, truly POC diagnostic that allows confident evidence-based decisions on treatment following a single patient visit is essential to accelerate the aim to end TB [46]. TB patient antibodies to MA have shown promise as a unique biomarker for active infection [18]. However, the low affinity of anti-MA antibodies [22] presents challenges to their detection in POC assays. This study demonstrates that MA-specific low-affinity antibodies can be detected using LFIA. To enable this unusual lipid-based LFIA use-case, the ‘MALIA’ test detects the displacement of recombinant monoclonal antibodies (gallibodies) bound to MA via a secondary antibody. 

The chicken-derived, recombinant monoclonal antibodies (gallibodies) were selected for their ability to bind to a purified, natural mixture of MAs [47] with ELISA [29]. Because affinity purification of anti-MA antibodies from serum is not possible due to low binding avidity, the gallibodies provided a unique opportunity to investigate whether and how the MA antigen is detected by anti-MA antibodies in/on whole mycobacteria. Whereas the gallibody failed to recognise MA in whole mycobacterial cells, the anti-*M. tuberculosis* antibody failed to recognise the immobilised reagent MA. This potentially implicates the absence of an accessible antigenic MA surface on the outer surface of the mycobacteria itself, or the undetectably low avidity of the antibodies to MA at the antibody dilution of 1:200. From these data (Figure 2), we conclude that whole cell detection by the anti-MA gallibodies is not practical. The gallibodies did not detect MA when presented in the context of fixed whole bacteria in ELISA. In contrast, the glycolipid antigen LAM, which is thought to be located beneath or partially beneath MA on the bacterial surface [48] is detectable on whole bacteria using an anti-LAM sandwich immunoassay [49]. Serum antibodies to MA are known to bind coated, purified MA [18,22,32], and the gallibodies were successfully displaced from commercially obtained, purified MA by serum antibodies. Taken together, this may suggest that the antigenic MA ‘surface’/epitope presented to the immune system is dissimilar to that on the surface of whole cultured bacteria. The lack of binding to an antigenic MA surface on whole bacteria could also be because of the obscuring of the MA by other cell wall components, or an insufficient concentration for detectable recognition by the gallibodies. Culture conditions and age are also known to heavily influence mycobacterial cell wall composition [33,47]. The MA antigen is defined by functional groups on the mero-chain and their stereochemical orientation [29]. However, the surface created by packed MAs is antigenic, rather than individual MAs [32]. The antigenicity of this surface is sensitive to the MA subclass composition [32] and may undergo conformational change by density change [50]. In the presence of other hexane-soluble components (in the crude extract), a more antigenic conformation may be produced by dilution of the extract (Figure 3). This is different to the binding of monoclonal antibodies to cholesterol monohydrate crystals that have been shown to have decreased antigenicity upon dilution [51]. Similarly, purified MA does not increase in antigenicity upon dilution (between 250–0.78 µg/mL)). 

Figure 2 and Figure 3 provide a significant contribution to the characterisation of the monoclonal anti-MA gallibodies and the understanding of MA as a biomarker antigen. The gallibodies are not able to bind to MA on whole bacteria grown under laboratory conditions but they did specifically bind mycobacterial extracts. Poor specificity of the gallibody itself would result in high false-positive results when used for diagnosis. The observed preferential binding of *M. tuberculosis* and *M. bovis* extracts was expected as the natural mixture of MAs used to select for the gallibodies was isolated from *M. tuberculosis*, which has identical MA subclasses but in different ratios compared to *M. bovis* [43]. 

The subclass specificity of the gallibodies reported previously [29] did not translate directly to mycobacterial differentiation as hypothesized. For example, *M. abscessus* does not contain methoxy MA [43] but was detected by both 18-1 and 18-2 gallibodies, which were previously shown to bind to only methoxy MA or mixtures containing it [29]. This may be due to induced antigenicity provided by the other elements present in the crude extracts. 

The straightforward detection of anti-MA antibodies as a biomarker for active TB is challenging due to cross-reactivity of naturally occurring [52,53] antibodies to cholesterol [22,54]. This challenge was first overcome by the use of real-time binding inhibition detection using a sensitive biosensor [18]. For the LFIA (MALIA, Figure 1), patient serum sample antibodies to MA compete with the monoclonal anti-MA gallibodies for binding MA on the test line. Active TB diagnosis is complicated by the high prevalence of latent TB infection [22] in countries with a high burden of TB and HIV. In particular, patients co-infected with TB and HIV tend to give false-negative anti-TB antibody results [7,18]. MALIA detects CD4 T-independent anti-lipid antibodies and so is expected not to suffer from this weakness [18,22,47]. To initially verify the displacement principle, samples were used from an experimental environment, from which latent TB, previous *M. tuberculosis* infection, as well as vaccination could be ruled out entirely.

The valency of the gallibodies (two) produces a lower avidity relative to the serum anti-MA antibodies, which are likely to contain multivalent IgM antibodies [19]. As MALIA requires displacement of the bound gallibodies by serum anti-MA antibodies, a range of available binding strengths and understanding of these is very useful.

It is reasonable to suppose that the gallibodies with the weakest binding to MA in ELISA, 18-1, 16-2 and 16-1 (Figure 3) would be the best displaced by the TB-positive samples. While gallibodies 16-1 and 18-1 were consistently well displaced, gallibody 16-2 was not, potentially explained by the influence of the more rigid scaffold structure, allowing better resistance to displacement. The 12-1 and 12-2 gallibodies were consistently shown to strongly bind to MA (ref [29], Figure 3 and with dark TB-negative test lines in Figure 4) and also showed poor displacement irrespective of the scaffold.

The poor distinction between TB-positive and TB-negative samples at higher gallibody concentrations (Figure 5) may be due to tighter packing of gallibody and potentially increased lateral binding between the gallibodies at higher concentrations making them harder to displace. The gallibodies are recombinantly expressed in human embryonic kidney cells and are likely to have some slightly misfolded/ hydrophobic regions to make them vulnerable to lateral binding.

This work enabled the selection of the most responsive gallibody: 16-1. Gallibody 16-1 does not bind the cholesteroid fold of MA, thought to be brought about by alpha and cis-keto MA subclasses, the latter two of which are not recognised by Gallibody 16-1 [29]. These features translated to specificity for mycobacteria, preferentially binding crude *M. tuberculosis* and *M. bovis* extracts in ELISA (Figure 3). The low sensitivity of 16-1 (ref [29] and Figure 3) enables the biggest displacement by serum anti-MA antibodies (Figure 4) compared to the other gallibodies. The more flexible scaffolds (16-1, 18-1 and 12-1) bind less strongly to MA than their more rigid counterparts (Figure 3) supporting the inference of Ranchod et al. [29] that the additional constantly heavy domain influences the dynamics of binding to the MA epitope.

The Fiji LFIA test line quantitation measures the ‘brightness’ (based on the red, green and blue colour values) of the pixels [41] and so is vulnerable to scan artefacts such as background and shadows (for example as in Figure 6). Detection of slight displacement (for example, Figure 4A, 12-1, 12-2 and 16-2) was not possible by eye so this quantitation was useful for the purpose of this work. However, user interpretation of a (perhaps only slightly) decreased signal is too difficult for robust field deployment. We envision the use of a low-cost reader such as that developed by Smith et al. [40] that will provide real-time connectivity and enable equipment-free application of the test. Significant signal variation observed between replicate tests on occasion (e.g., Figure 4A, 18-2*) points to the known challenges of quality control that LFIAs face [12].

Currently, MALIA requires more than an hour to complete due to multiple flow steps that decrease in speed. A different flow design and thicker wick could be applied to reduce this time. To make the diagnostic more robust and user-friendly, the workflow will need to be simplified. Incorporation of a reliable negative control signal is essential, as complete signal removal is unlikely to be consistently observed across patients. Signal reduction cut-off values will need to be determined using a large sample size for use with the low-cost reader. Membrane-based blood separation will allow whole, fingerstick blood to be used (allowing equipment-free ease of specimen collection), similar to HIV rapid tests [55].

High-affinity antibody/antigen pairs are sought after for sensitive LFIA development [49,56]. However, the detection of displacement of gallibodies by low-affinity TB-positive serum antibodies to MA in LFIA has been demonstrated in this work. This proof-of-concept device remains to be engineered to provide a REASSURED [12] device for the detection of low-affinity anti-lipid antibodies in patient blood samples at the POC. As anti-lipid antibodies have been shown to reduce with successful treatment [16,17], MALIA may also be applicable to the treatment monitoring as well as the non-sputum-based biomarker and triage TPPs. 

## Data Availability

The original contributions presented in the study are included in the article/Supplementary Material, further inquiries can be directed to the corresponding author.

## Supplementary Materials

The following supporting information can be downloaded at: https://www.mdpi.com/article/10.3390/tropicalmed9110269/s1, Table S1: CFU counts of organs of TB-positive guinea pigs; Figure S1: Yellow rectangle in the test line is the region of interest selection for Fiji quantitation; Figure S2: Qualitative thin layer chromatography analysis of mycobacterial extract; Table S2: Rf values of presumed MA spots in Figure S2; Figure S3: Control tests sections with all gallibodies; Table S3: Quantified MA test line values for buffer-only tests with all gallibodies; Figure S4: Control test sections at 4 concentrations of gallibody 16-1; Table S4: Quantified MA test line values for buffer-only tests at 4 concentrations of gallibody 16-1. Figure S5: Control test sections for human serum experiment; Table S5: Quantified MA test line values for control tests for human serum experiment.
