# Supplementary material for: Application of Monoclonal Anti-Mycolate Antibodies in Serological Diagnosis of Tuberculosis
Source: Trop Med Infect Dis. 2024 Nov 6;9(11):269. doi: 10.3390/tropicalmed9110269 (PMC11598376; doi:10.3390/tropicalmed9110269)
Supplement: Supplementary file 1 [file tropicalmed-09-00269-s001.zip › tropicalmed-3254254-supplementary.pdf]

S. Supplementary Material

Guinea pig organ *M. tuberculosis* CFU count

TB positive animals were confirmed by homogenising the lungs and spleen in PBS, plating serial dilutions of these on 7H11 agar plates and enumerating the viable bacteria by CFU counts given in **Error! Reference source not found.**.

Table S1. CFU counts of organs of TB positive guinea pigs

Animal	Lung CFU	Spleen CFU
x	16 200 000	13 500
y	1 323 000	2 250
z	621 000	0

Region of interest selection for FIJI quantitation

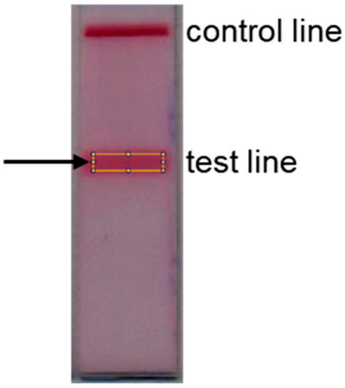

Figure S1. Yellow rectangle in the test line is the region of interest selection for FIJI quantitation.

Thin Layer chromatography

The extracts were analysed using thin layer chromatography. Extracts and controls were spotted on aluminium backed silica plates. Two spots of 4 µL each were placed on top of each other using micropipettes (Blaubrand 708707) on a marked pencil line 1 cm from the edge of the membrane. The spotted and dried silica membrane was placed in an equilibrated tank containing a solvent mixture of chloroform, acetone and acetic acid at a ratio of 70:6:1. Once the solvent reached about 2 cm from the top of the membrane it was removed from the tank and the solvent front marked with pencil. The membrane was briefly dried with a heat gun (Master Proheat PH-2100) before dipping in a solution of 10% (w/v) phosphomolybdic acid in ethanol and charring using the heat gun. The membranes were imaged using a ChemiDoc XRS+ (Bio-Rad, Hemel Hempstead, UK) running ImageLab. Cropped 'lanes' of the imaged membranes are shown. Retention factor (Rf) values are indicated in table S2.

Replicate numbers (1, 2 or 3) for the *M. abscessus* extracts are not matched to the ELISA replicate numbers as plates prepared from the same suspension were used for the extract.

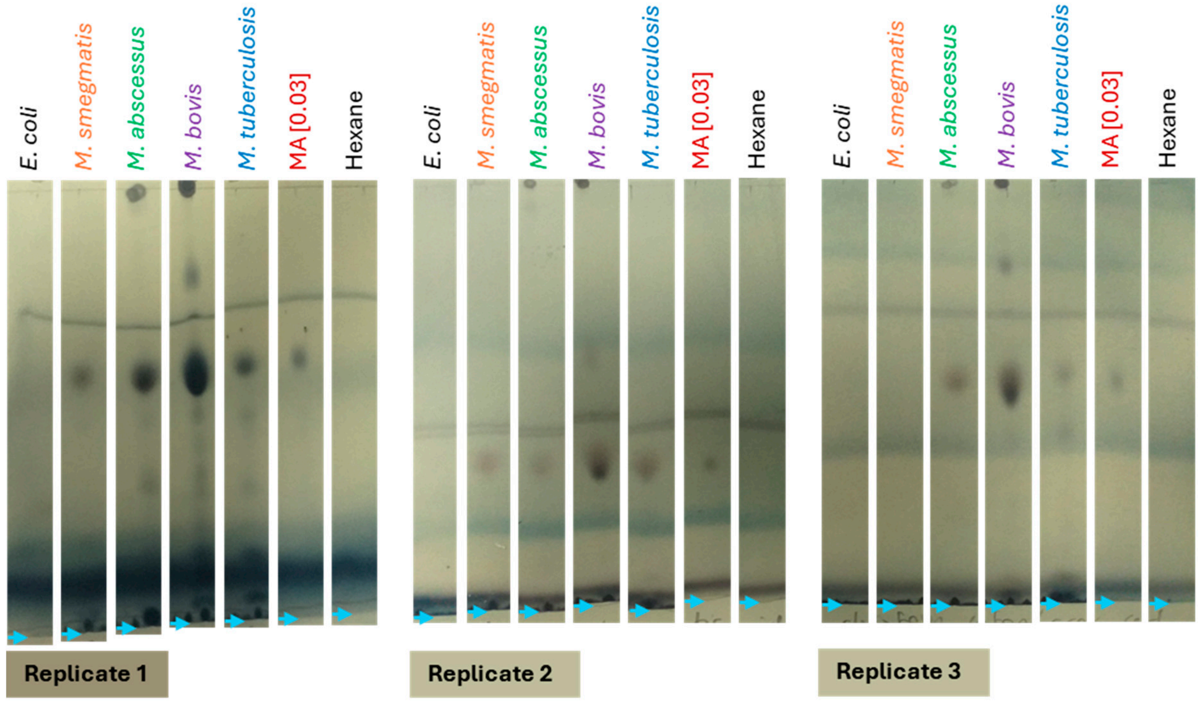

Figure S2. Qualitative thin layer chromatography analysis of mycobacterial extracts. An 8 µL volume of crude hexane extracts of bacteria, purified commercial mycolic acid (MA) at 31.25 µg/mL or hexane was spotted on aluminium backed silica using micropipettes. Silica plates were run in a solvent mixture of chloroform, acetone and acetic acid at a ratio of 70:6:1. Phosphomolybdic acid (10% w/v in ethanol) was used as the stain. The solvent front is indicated with blue arrows. Lanes are cropped from same image, in replicate 2 *M. bovis* and *M. tuberculosis* lanes are swapped to allow consistent labelling.

Table S2. Rf values of presumed MA spots in Figure S2

**Replicate #**	**1**	**2**	**3**
E. coli	0.00	0.00	0.00
M. smegmatis	0.42	0.66	0.00
M. abscessus	0.44	0.65	0.48
M. bovis	0.44	0.67	0.49
M. tuberculosis	0.41	0.67	0.46
**MA**	0.40	0.67	0.47
**Hexane**	0.00	0.00	0.00

No spots are visible in the hexane or *E. coli* extract lanes. Spots migrating to the same/similar degree as the control mycolic acid are visible for all the other extracts except the *M. smegmatis* extract for replicate 3. Rf values for these spots are shown in Table S-2. The *M. bovis* extracts contain several other visible spots to varying degrees across the replicates.

LFIA controls

To confirm the MA test line signal in the absence of interference from serum components tests were performed with all the gallibodies but without serum (Figure S.3A). To confirm no non-specific binding occurred on the test line bovine serum albumin was substituted for the gallibody (Figure S.3B).

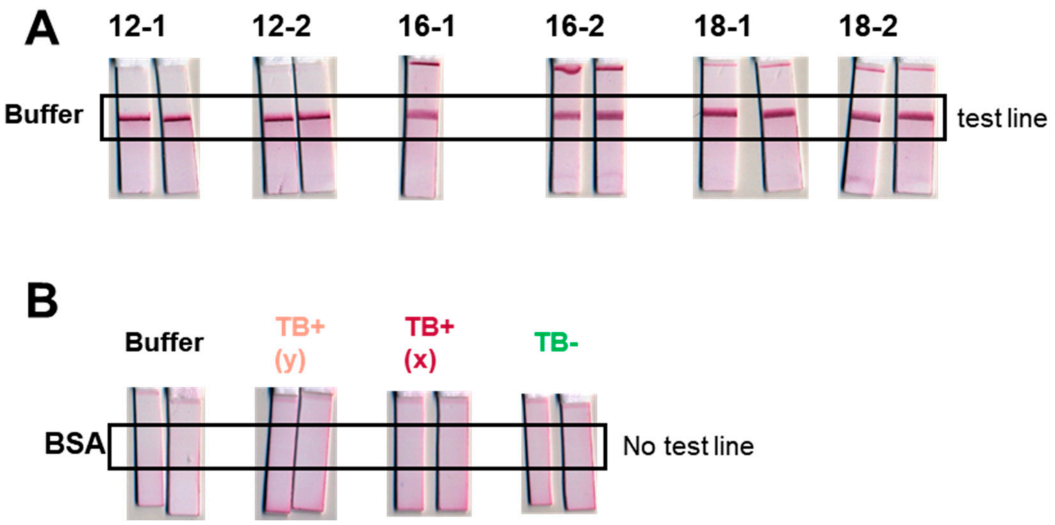

Figure S3. Control test sections with all gallibodies. Scanned test sections. Gallibodies (A) at 0.06 mg/mL or (B bovine serum albumin (BSA at 1 mg/mL diluted in membrane blocker was flowed on tests striped with anti-chicken IgG (Fc antibody (top line at 0.25 mg/mL and mycolic acid (bottom line at 3 x 0.5 mg/mL for ~15 minutes. Subsequently, 50 µL of (A membrane blocker containing rheumatoid factor interference blocker (1 mg/mL or (B 10% guinea pig serum (pooled serum from TB negative animals – 'TB-', two TB positive animals – 'TB+ (x' and 'TB+ (y diluted in membrane blocker containing rheumatoid factor interference blocker (1 mg/mL was flowed until absorbed. 'Buffer' tests excluded serum. Finally, 50 µL of membrane blocker containing 3 µL of anti-chicken-gold conjugate was flowed. Cropped scans of the test section of duplicate tests are shown. Test line indicated by black box.

The MA test line intensities from Figure S.3A was quantified using FIJI software, results are given in Table S-3. Darker test lines give lower values.

Table S3. Quantified MA test line values for control tests with all gallibodies

Gallibody	Replicate 1	Replicate 2
12-1	69.846	76.89
12-2	75.41	73.588
16-1	103.242	*
16-2	83.956	100.61
18-1	77.819	76.225
18-2	82.44	97.451

* only 1 test was performed

Due to the differing sensitivities of the gallibodies varying MA test line signal intensities (Figure S.3A and Table S-3) was expected. No signal was observed in the absence of gallibody (Figure S.3B) as expected.

Gallibody 16-1 was titrated to investigate the effect of the concentration of the gallibody on the displacement. As before, tests were performed in the absence of serum to determine MA test line signal. Tests are shown in Figure S.4 and quantified MA test line signal intensities are provided in Table S-4.

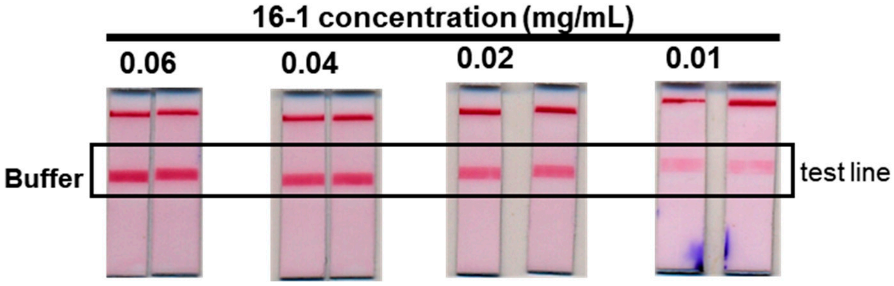

Figure S4. Control test sections at 4 concentrations of gallibody 16-1 Gallibody 16-1 at 0.06, 0.04, 0.02 and 0.01 mg/mL diluted in membrane blocker was flowed on tests striped with anti-chicken IgG (Fc) antibody (top line) at 0.25 mg/mL and mycolic acid (bottom line) at 3 x 0.5 mg/mL for ~15 minutes. Subsequently, 50 µL of membrane blocker containing Rheumatoid factor inhibition blocker (1 mg/mL) was flowed until absorbed, followed by 50 µL of membrane blocker containing 3 µL of anti-chicken-gold conjugate. Cropped scans of the test section of duplicate tests are shown. Test line indicated by black box.

Table S4. Quantified MA test line values for control tests at 4 concentrations of gallibody 16-1

Concentration	Replicate 1	Replicate 2
0.06 mg/mL	70.69	75.181
0.04 mg/mL	82.924	80.329
0.02 mg/mL	99.781	100.157
0.01 mg/mL	132.229	134.271

As expected, MA test line intensity reduces with reducing gallibody concentration.

For the human serum experiment tests were also performed in the absence of serum to determine MA test line signal. Tests are shown in Figure S.5 and quantified MA test line signal intensities are provided in Table S-5.

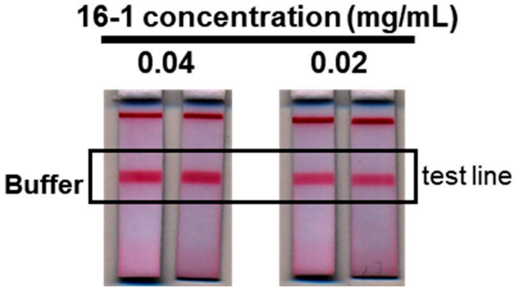

Figure S5. Control test sections for human serum experiment Gallibody 16-1 at 0.04 and 0.02 mg/mL diluted in membrane blocker was flowed on tests striped with anti-chicken IgG (Fc) antibody (top line) at 0.25 mg/mL and mycolic acid (bottom line) at 3 x 0.5 mg/mL for ~15 minutes. Subsequently, 50 µL of membrane blocker containing Rheumatoid factor inhibition blocker (1 mg/mL) was flowed until absorbed. Tests were then developed with 50 µL of membrane blocker containing 3 µL of anti-chicken-

gold conjugate. Cropped scans of the test section of duplicate tests are shown. Test line indicated by black box.

Table S5. Quantified MA test line values for control tests for human serum experiment

Concentration	Replicate 1	Replicate 2
0.04 mg/mL	86.926	81.859
0.02 mg/mL	100.226	98.330
